# Crystal structure of 3-(di­ethyl­amino)­phenol

**DOI:** 10.1107/S2056989015024226

**Published:** 2015-12-24

**Authors:** James A. Golen, Kyle J. McDonald, David R. Manke

**Affiliations:** a Department of Chemistry and Biochemistry, University of Massachusetts Dartmouth, 285 Old Westport Road, North Dartmouth, MA 02747, USA; b Department of Science & Math, Massasoit Community College, 1 Massasoit Boulevard, Brockton, MA 02302, USA

**Keywords:** crystal structure, hydrogen bonding, phenols

## Abstract

The title compound, C_10_H_15_NO, has two mol­ecules in the asymmetric unit. Each mol­ecule has a near-planar C_8_NO unit excluding H atoms and the terminal methyl groups on the di­ethyl­amino groups, with mean deviations from planarity of 0.036 and 0.063 Å. In the crystal, hydrogen bonding leads to four-membered O—H⋯O—H⋯O—H·· rings. No π–π inter­actions were observed in the structure.

## Related literature   

For the structure of 3-amino­phenol, see: Allen *et al.* (1997 ▸). For the structure of similar 3-amino­phenols, see: Xu *et al.* (2004 ▸); Suchetan *et al.* (2014 ▸). For background, see: McDonald *et al.* (2015 ▸); Mills-Robles *et al.* (2015 ▸); Nguyen *et al.* (2015 ▸).
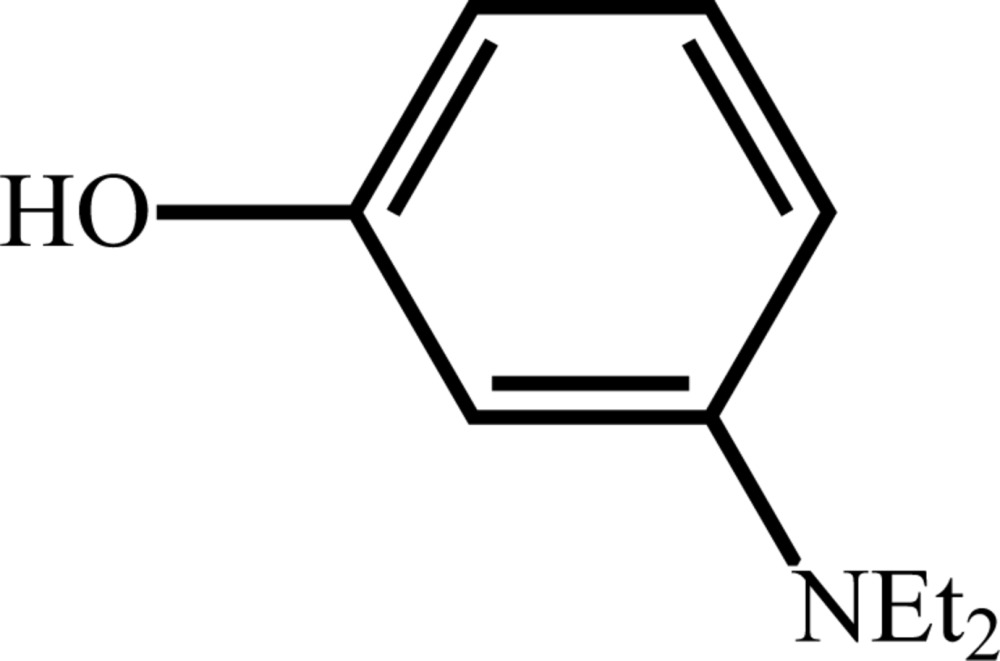


## Experimental   

### Crystal data  

C_10_H_15_NO
*M* *_r_* = 165.23
Orthorhombic, 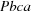

*a* = 14.5166 (17) Å
*b* = 15.9102 (18) Å
*c* = 16.0527 (18) Å
*V* = 3707.6 (7) Å^3^
*Z* = 16
Cu *K*α radiation
μ = 0.60 mm^−1^
*T* = 120 K
0.25 × 0.2 × 0.1 mm

### Data collection  

Bruker D8 Venture CMOS diffractometer
Absorption correction: multi-scan (*SADABS*; Bruker, 2014 ▸) *T*_min_ = 0.679, *T*_max_ = 0.753
21122 measured reflections
3398 independent reflections
2633 reflections with *I* > 2σ(*I*)
*R*_int_ = 0.090

### Refinement  

*R*[*F* ^2^ > 2σ(*F* ^2^)] = 0.042
*wR*(*F* ^2^) = 0.107
*S* = 1.02
3398 reflections
228 parameters
2 restraints
H atoms treated by a mixture of independent and constrained refinement
Δρ_max_ = 0.19 e Å^−3^
Δρ_min_ = −0.20 e Å^−3^

### 

Data collection: *APEX2* (Bruker, 2014 ▸); cell refinement: *SAINT* (Bruker, 2014 ▸); data reduction: *SAINT*; program(s) used to solve structure: *SHELXS97* (Sheldrick, 2008 ▸); program(s) used to refine structure: *SHELXL2014* (Sheldrick, 2015 ▸); molecular graphics: *OLEX2* (Dolomanov *et al.*, 2009 ▸); software used to prepare material for publication: *OLEX2* and *publCIF* (Westrip, 2010 ▸).

## Supplementary Material

Crystal structure: contains datablock(s) I. DOI: 10.1107/S2056989015024226/ff2147sup1.cif

Structure factors: contains datablock(s) I. DOI: 10.1107/S2056989015024226/ff2147Isup2.hkl

Supporting information file. DOI: 10.1107/S2056989015024226/ff2147Isup3.cml

**Fig. 1.**
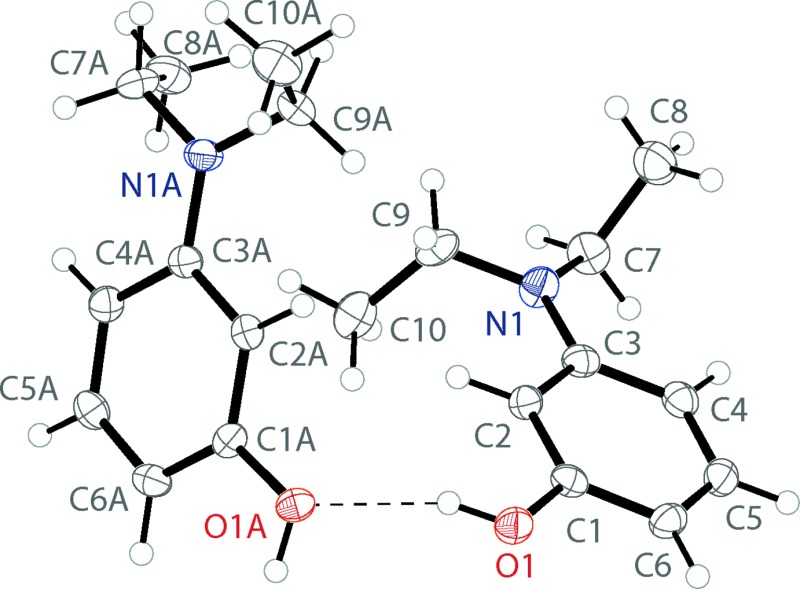
Molecular structure of the title compound, showing the atom-labelling scheme. Displacement ellipsoids are drawn at the 50% probability level. H atoms are drawn as spheres of arbitrary radius.

. DOI: 10.1107/S2056989015024226/ff2147fig1.tif
Mol­ecular structure of the title compound, showing the atom-labelling scheme. Displacement ellipsoids are drawn at the 50% probability level. H atoms are drawn as spheres of arbitrary radius.

**Fig. 2.**
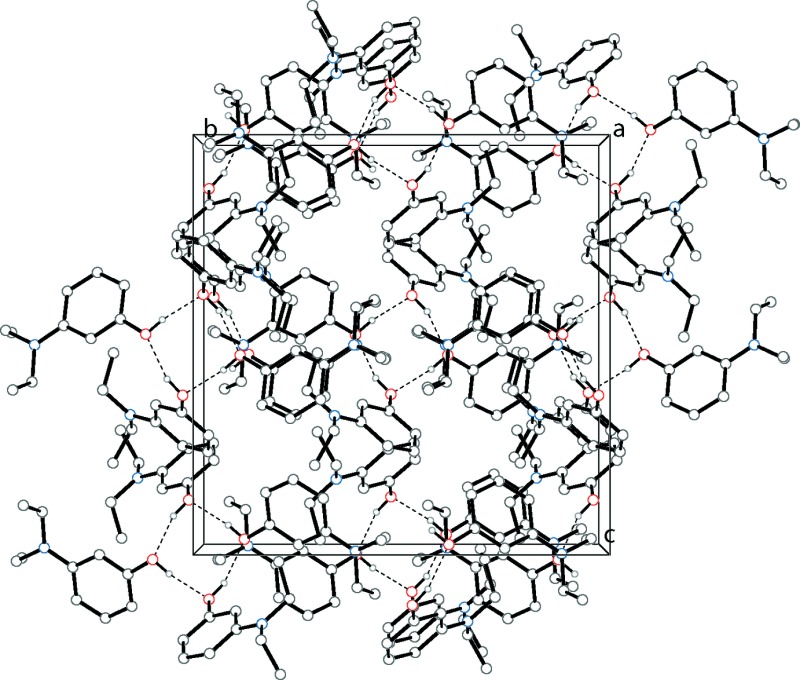
Molecular packing of the title compound with hydrogen bonding shown as dashed lines.

. DOI: 10.1107/S2056989015024226/ff2147fig2.tif
Mol­ecular packing of the title compound with hydrogen bonding shown as dashed lines.

CCDC reference: 1442843

Additional supporting information:  crystallographic information; 3D view; checkCIF report

## Figures and Tables

**Table 1:** Hydrogen-bond geometry (Å, °)

*D*—H⋯*A*	*D*—H	H⋯*A*	*D*⋯*A*	*D*—H⋯*A*
O1—H1⋯O1*A*	0.86 (1)	1.92 (1)	2.7445 (16)	160 (2)
O1*A*—H1*A*⋯O1^i^	0.86 (1)	1.91 (1)	2.7599 (16)	170 (2)

Symmetry code: (i) 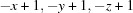.

## supplementary crystallographic information

### S1. Comment

Herein we report the structure of 3-(di­ethyl­amino)­phenol as part of a continuing collaboration between UMass Darmouth and Massasoit Community College to examine the solid state structure of aromatic alcohols (McDonald *et al.*, 2015; Mills-Robles *et al.*, 2015; Nguyen *et al.*, 2015). Hydrogen bonding in the title compound leads to four-membered O1–H1···O1A–H1A···O1–H1·· rings. The molecules with the greatest structural similarity whose solid state structure have been reported all demonstrate hydrogen bonding with different acceptors. The parent 3-amino­phenol (Allen *et al.*, 1997) and 3-(1*H*-1,2,4-triazol-4-yl)phenol (Xu *et al.*, 2004) both instead demonstrate O–H···N hydrogen bonding. The structure of *N*-(3-hy­droxy­phenyl)­succinimide possesses O–H···O inter­actions with carbonyl oxygen atoms (Suchetan *et al.*, 2014) rather than phenol only inter­actions.

The molecular structure of the title compound has two molecules in the asymmetric unit. Each molecule has a near planar C_8_NO unit excluding H atoms and the terminal methyls on the di­ethyl­amino groups (C8, C10 and C8A, C10A). This unit for the molecule containing O1 has a mean deviations from planarity of 0.036 Å and the C_8_NO unit for molecule containing O1A has a mean deviation from planarity of 0.063 Å. No π-π inter­actions were observed in the structure. The packing for the title compound indicating hydrogen bonding is shown in Figure 2.

### S2. Experimental

Crystals suitable for X-ray diffraction studies were selected from a commercial sample (Aldrich).

### S3. Refinement

All non-hydrogen atoms were refined anisotropically (*XL*) by full matrix least squares on F^2^. Hydrogen atoms H1 and H1A were found from a Fourier difference map, and refined with a fixed distance of 0.86 (0.01) Å and isotropic displacement parameters of 1.50 times *U*_eq_ of the parent O atoms. The remaining hydrogen atoms were placed in calculated positions and then refined with a riding model with C–H lengths of 0.95 Å (*sp*^2^) and 0.98 Å (*sp*^3^) with isotropic displacement parameters set to 1.20 (*sp*^2^) and 1.50 (*sp*^3^) times *U*_eq_ of the parent C atom.

### Crystal data

**Table:**

C_10_H_15_NO	*F*(000) = 1440
*M**_r_* = 165.23	*D*_x_ = 1.184 Mg m^−^^3^
Orthorhombic, *P**b**c**a*	Cu *K*α radiation, λ = 1.54178 Å
Hall symbol: -P 2ac 2ab	Cell parameters from 8014 reflections
*a* = 14.5166 (17) Å	θ = 5.0–68.1°
*b* = 15.9102 (18) Å	µ = 0.60 mm^−^^1^
*c* = 16.0527 (18) Å	*T* = 120 K
*V* = 3707.6 (7) Å^3^	SHARD, colourless
*Z* = 16	0.25 × 0.2 × 0.1 mm

### Data collection

**Table:**

Bruker D8 Venture CMOS diffractometer	3398 independent reflections
Radiation source: Cu	2633 reflections with *I* > 2σ(*I*)
HELIOS MX monochromator	*R*_int_ = 0.090
φ and ω scans	θ_max_ = 68.4°, θ_min_ = 5.0°
Absorption correction: multi-scan (*SADABS*; Bruker, 2014)	*h* = −17→17
*T*_min_ = 0.679, *T*_max_ = 0.753	*k* = −18→19
21122 measured reflections	*l* = −11→19

### Refinement

**Table:**

Refinement on *F*^2^	Hydrogen site location: mixed
Least-squares matrix: full	H atoms treated by a mixture of independent and constrained refinement
*R*[*F*^2^ > 2σ(*F*^2^)] = 0.042	*w* = 1/[σ^2^(*F*_o_^2^) + (0.0402*P*)^2^ + 1.2567*P*] where *P* = (*F*_o_^2^ + 2*F*_c_^2^)/3
*wR*(*F*^2^) = 0.107	(Δ/σ)_max_ < 0.001
*S* = 1.02	Δρ_max_ = 0.19 e Å^−^^3^
3398 reflections	Δρ_min_ = −0.20 e Å^−^^3^
228 parameters	Extinction correction: *SHELXL*, Fc^*^=kFc[1+0.001xFc^2^λ^3^/sin(2θ)]^-1/4^
2 restraints	Extinction coefficient: 0.0024 (2)

### Special details

**Table:**

Experimental. Absorption correction: *SADABS2014*/4 (Bruker,2014/4) was used for absorption correction. wR2(int) was 0.1095 before and 0.0838 after correction. The Ratio of minimum to maximum transmission is 0.9012. The λ/2 correction factor is 0.00150.
Geometry. All e.s.d.'s (except the e.s.d. in the dihedral angle between two l.s. planes) are estimated using the full covariance matrix. The cell e.s.d.'s are taken into account individually in the estimation of e.s.d.'s in distances, angles and torsion angles; correlations between e.s.d.'s in cell parameters are only used when they are defined by crystal symmetry. An approximate (isotropic) treatment of cell e.s.d.'s is used for estimating e.s.d.'s involving l.s. planes.

### Fractional atomic coordinates and isotropic or equivalent isotropic displacement parameters (Å^2^)

**Table:**

	*x*	*y*	*z*	*U*_iso_*/*U*_eq_	
O1	0.53496 (8)	0.52600 (7)	0.61994 (7)	0.0255 (3)	
H1	0.5319 (14)	0.5635 (10)	0.5812 (10)	0.038*	
N1	0.82785 (10)	0.66193 (9)	0.66954 (9)	0.0271 (3)	
C1	0.61885 (11)	0.53018 (10)	0.66024 (10)	0.0209 (3)	
C2	0.68001 (11)	0.59514 (10)	0.64446 (10)	0.0211 (3)	
H2	0.6639	0.6379	0.6058	0.025*	
C3	0.76619 (12)	0.59821 (9)	0.68541 (10)	0.0214 (4)	
C4	0.78585 (12)	0.53347 (10)	0.74327 (10)	0.0234 (4)	
H4	0.8423	0.5342	0.7732	0.028*	
C5	0.72339 (12)	0.46927 (10)	0.75652 (10)	0.0249 (4)	
H5	0.7385	0.4260	0.7950	0.030*	
C6	0.63954 (12)	0.46591 (10)	0.71552 (10)	0.0250 (4)	
H6	0.5976	0.4211	0.7249	0.030*	
C7	0.92033 (12)	0.66198 (11)	0.70474 (11)	0.0281 (4)	
H7A	0.9631	0.6884	0.6644	0.034*	
H7B	0.9405	0.6031	0.7130	0.034*	
C8	0.92687 (14)	0.70839 (12)	0.78718 (12)	0.0367 (5)	
H8A	0.9910	0.7089	0.8061	0.055*	
H8B	0.8885	0.6800	0.8287	0.055*	
H8C	0.9053	0.7663	0.7799	0.055*	
C9	0.80758 (12)	0.72813 (10)	0.60980 (11)	0.0275 (4)	
H9A	0.8460	0.7778	0.6230	0.033*	
H9B	0.7422	0.7448	0.6159	0.033*	
C10	0.82475 (13)	0.70291 (11)	0.52001 (11)	0.0323 (4)	
H10A	0.8167	0.7520	0.4838	0.048*	
H10B	0.7809	0.6590	0.5038	0.048*	
H10C	0.8877	0.6814	0.5144	0.048*	
O1A	0.50614 (8)	0.61366 (7)	0.47501 (7)	0.0254 (3)	
N1A	0.63804 (10)	0.88712 (8)	0.50028 (8)	0.0238 (3)	
C1A	0.55769 (11)	0.67627 (9)	0.43824 (10)	0.0196 (3)	
H1A	0.4968 (13)	0.5730 (9)	0.4404 (10)	0.029*	
C2A	0.57128 (10)	0.74811 (9)	0.48544 (9)	0.0186 (3)	
H2A	0.5458	0.7517	0.5398	0.022*	
C3A	0.62260 (11)	0.81588 (9)	0.45334 (9)	0.0188 (3)	
C4A	0.65707 (11)	0.80848 (10)	0.37146 (10)	0.0214 (4)	
H4A	0.6905	0.8536	0.3473	0.026*	
C5A	0.64237 (11)	0.73569 (10)	0.32641 (10)	0.0234 (4)	
H5A	0.6669	0.7317	0.2717	0.028*	
C6A	0.59298 (11)	0.66813 (10)	0.35830 (10)	0.0228 (4)	
H6A	0.5837	0.6184	0.3266	0.027*	
C7A	0.68674 (12)	0.95955 (10)	0.46599 (11)	0.0249 (4)	
H7AA	0.6678	1.0105	0.4969	0.030*	
H7AB	0.6681	0.9671	0.4071	0.030*	
C8A	0.79083 (12)	0.95156 (11)	0.47007 (11)	0.0303 (4)	
H8AA	0.8192	1.0041	0.4512	0.045*	
H8AB	0.8109	0.9053	0.4340	0.045*	
H8AC	0.8096	0.9401	0.5276	0.045*	
C9A	0.61651 (12)	0.89193 (10)	0.58880 (10)	0.0248 (4)	
H9AA	0.6664	0.9230	0.6175	0.030*	
H9AB	0.6150	0.8343	0.6120	0.030*	
C10A	0.52551 (13)	0.93469 (12)	0.60712 (12)	0.0358 (5)	
H10D	0.5179	0.9405	0.6675	0.054*	
H10E	0.4750	0.9008	0.5846	0.054*	
H10F	0.5248	0.9904	0.5812	0.054*	

### Atomic displacement parameters (Å^2^)

**Table:**

	*U*^11^	*U*^22^	*U*^33^	*U*^12^	*U*^13^	*U*^23^
O1	0.0246 (7)	0.0244 (6)	0.0276 (6)	−0.0063 (5)	−0.0026 (5)	0.0020 (5)
N1	0.0210 (8)	0.0279 (7)	0.0323 (8)	−0.0061 (6)	−0.0042 (6)	0.0053 (6)
C1	0.0211 (8)	0.0223 (7)	0.0194 (8)	−0.0007 (6)	0.0019 (6)	−0.0043 (6)
C2	0.0241 (9)	0.0198 (7)	0.0195 (8)	−0.0003 (6)	0.0016 (6)	0.0001 (6)
C3	0.0219 (9)	0.0206 (8)	0.0216 (8)	0.0001 (6)	0.0037 (6)	−0.0036 (6)
C4	0.0244 (9)	0.0252 (8)	0.0207 (8)	0.0035 (7)	−0.0003 (6)	−0.0032 (7)
C5	0.0321 (10)	0.0226 (8)	0.0201 (8)	0.0048 (7)	0.0047 (7)	0.0007 (6)
C6	0.0301 (10)	0.0202 (8)	0.0246 (8)	−0.0022 (7)	0.0063 (7)	0.0005 (7)
C7	0.0199 (9)	0.0336 (9)	0.0309 (9)	−0.0044 (7)	0.0002 (7)	0.0002 (7)
C8	0.0406 (12)	0.0394 (10)	0.0300 (10)	−0.0106 (9)	−0.0043 (8)	−0.0004 (8)
C9	0.0240 (9)	0.0203 (8)	0.0381 (10)	−0.0041 (7)	−0.0009 (7)	0.0027 (7)
C10	0.0258 (10)	0.0330 (9)	0.0379 (10)	−0.0002 (8)	0.0024 (8)	0.0072 (8)
O1A	0.0278 (7)	0.0209 (6)	0.0274 (6)	−0.0078 (5)	−0.0013 (5)	0.0007 (5)
N1A	0.0287 (8)	0.0206 (7)	0.0222 (7)	−0.0052 (6)	0.0038 (6)	−0.0041 (5)
C1A	0.0147 (8)	0.0192 (7)	0.0249 (8)	−0.0013 (6)	−0.0026 (6)	0.0033 (6)
C2A	0.0155 (8)	0.0218 (8)	0.0185 (8)	0.0015 (6)	−0.0001 (6)	0.0004 (6)
C3A	0.0160 (8)	0.0192 (7)	0.0211 (8)	0.0004 (6)	−0.0022 (6)	−0.0006 (6)
C4A	0.0192 (9)	0.0227 (8)	0.0223 (8)	−0.0026 (6)	0.0002 (6)	0.0015 (6)
C5A	0.0218 (9)	0.0294 (8)	0.0190 (8)	0.0006 (7)	0.0017 (6)	−0.0020 (7)
C6A	0.0222 (9)	0.0220 (8)	0.0243 (8)	0.0010 (6)	−0.0034 (7)	−0.0055 (6)
C7A	0.0263 (9)	0.0163 (7)	0.0319 (9)	−0.0037 (7)	0.0029 (7)	−0.0027 (6)
C8A	0.0281 (10)	0.0327 (9)	0.0302 (9)	−0.0079 (7)	0.0018 (7)	−0.0062 (7)
C9A	0.0271 (10)	0.0269 (8)	0.0202 (8)	−0.0015 (7)	−0.0025 (7)	−0.0050 (6)
C10A	0.0320 (11)	0.0408 (10)	0.0348 (10)	0.0041 (9)	0.0066 (8)	−0.0080 (8)

### Geometric parameters (Å, º)

**Table:**

O1—H1	0.863 (9)	O1A—C1A	1.3786 (19)
O1—C1	1.381 (2)	O1A—H1A	0.863 (9)
N1—C3	1.376 (2)	N1A—C3A	1.379 (2)
N1—C7	1.457 (2)	N1A—C7A	1.460 (2)
N1—C9	1.455 (2)	N1A—C9A	1.457 (2)
C1—C2	1.386 (2)	C1A—C2A	1.385 (2)
C1—C6	1.387 (2)	C1A—C6A	1.388 (2)
C2—H2	0.9500	C2A—H2A	0.9500
C2—C3	1.414 (2)	C2A—C3A	1.408 (2)
C3—C4	1.416 (2)	C3A—C4A	1.411 (2)
C4—H4	0.9500	C4A—H4A	0.9500
C4—C5	1.382 (2)	C4A—C5A	1.382 (2)
C5—H5	0.9500	C5A—H5A	0.9500
C5—C6	1.385 (2)	C5A—C6A	1.390 (2)
C6—H6	0.9500	C6A—H6A	0.9500
C7—H7A	0.9900	C7A—H7AA	0.9900
C7—H7B	0.9900	C7A—H7AB	0.9900
C7—C8	1.518 (2)	C7A—C8A	1.518 (2)
C8—H8A	0.9800	C8A—H8AA	0.9800
C8—H8B	0.9800	C8A—H8AB	0.9800
C8—H8C	0.9800	C8A—H8AC	0.9800
C9—H9A	0.9900	C9A—H9AA	0.9900
C9—H9B	0.9900	C9A—H9AB	0.9900
C9—C10	1.517 (3)	C9A—C10A	1.515 (2)
C10—H10A	0.9800	C10A—H10D	0.9800
C10—H10B	0.9800	C10A—H10E	0.9800
C10—H10C	0.9800	C10A—H10F	0.9800
			
C1—O1—H1	110.5 (14)	C1A—O1A—H1A	110.6 (13)
C3—N1—C7	121.88 (14)	C3A—N1A—C7A	121.42 (13)
C3—N1—C9	121.59 (14)	C3A—N1A—C9A	122.76 (13)
C9—N1—C7	116.22 (14)	C9A—N1A—C7A	115.48 (13)
O1—C1—C2	121.02 (14)	O1A—C1A—C2A	116.05 (14)
O1—C1—C6	117.09 (14)	O1A—C1A—C6A	121.93 (14)
C2—C1—C6	121.88 (15)	C2A—C1A—C6A	122.02 (14)
C1—C2—H2	119.7	C1A—C2A—H2A	119.8
C1—C2—C3	120.50 (15)	C1A—C2A—C3A	120.46 (14)
C3—C2—H2	119.7	C3A—C2A—H2A	119.8
N1—C3—C2	120.99 (14)	N1A—C3A—C2A	121.02 (14)
N1—C3—C4	121.75 (15)	N1A—C3A—C4A	121.31 (14)
C2—C3—C4	117.26 (15)	C2A—C3A—C4A	117.67 (14)
C3—C4—H4	119.8	C3A—C4A—H4A	119.9
C5—C4—C3	120.41 (16)	C5A—C4A—C3A	120.17 (15)
C5—C4—H4	119.8	C5A—C4A—H4A	119.9
C4—C5—H5	118.9	C4A—C5A—H5A	118.8
C4—C5—C6	122.14 (16)	C4A—C5A—C6A	122.35 (15)
C6—C5—H5	118.9	C6A—C5A—H5A	118.8
C1—C6—H6	121.1	C1A—C6A—C5A	117.31 (14)
C5—C6—C1	117.77 (15)	C1A—C6A—H6A	121.3
C5—C6—H6	121.1	C5A—C6A—H6A	121.3
N1—C7—H7A	108.9	N1A—C7A—H7AA	108.9
N1—C7—H7B	108.9	N1A—C7A—H7AB	108.9
N1—C7—C8	113.32 (15)	N1A—C7A—C8A	113.57 (14)
H7A—C7—H7B	107.7	H7AA—C7A—H7AB	107.7
C8—C7—H7A	108.9	C8A—C7A—H7AA	108.9
C8—C7—H7B	108.9	C8A—C7A—H7AB	108.9
C7—C8—H8A	109.5	C7A—C8A—H8AA	109.5
C7—C8—H8B	109.5	C7A—C8A—H8AB	109.5
C7—C8—H8C	109.5	C7A—C8A—H8AC	109.5
H8A—C8—H8B	109.5	H8AA—C8A—H8AB	109.5
H8A—C8—H8C	109.5	H8AA—C8A—H8AC	109.5
H8B—C8—H8C	109.5	H8AB—C8A—H8AC	109.5
N1—C9—H9A	108.8	N1A—C9A—H9AA	108.9
N1—C9—H9B	108.8	N1A—C9A—H9AB	108.9
N1—C9—C10	113.69 (14)	N1A—C9A—C10A	113.58 (15)
H9A—C9—H9B	107.7	H9AA—C9A—H9AB	107.7
C10—C9—H9A	108.8	C10A—C9A—H9AA	108.9
C10—C9—H9B	108.8	C10A—C9A—H9AB	108.9
C9—C10—H10A	109.5	C9A—C10A—H10D	109.5
C9—C10—H10B	109.5	C9A—C10A—H10E	109.5
C9—C10—H10C	109.5	C9A—C10A—H10F	109.5
H10A—C10—H10B	109.5	H10D—C10A—H10E	109.5
H10A—C10—H10C	109.5	H10D—C10A—H10F	109.5
H10B—C10—H10C	109.5	H10E—C10A—H10F	109.5
			
O1—C1—C2—C3	−179.26 (14)	O1A—C1A—C2A—C3A	−179.69 (14)
O1—C1—C6—C5	−179.92 (14)	O1A—C1A—C6A—C5A	178.67 (14)
N1—C3—C4—C5	−178.55 (15)	N1A—C3A—C4A—C5A	178.57 (15)
C1—C2—C3—N1	179.29 (15)	C1A—C2A—C3A—N1A	−178.81 (15)
C1—C2—C3—C4	−1.0 (2)	C1A—C2A—C3A—C4A	1.6 (2)
C2—C1—C6—C5	1.4 (2)	C2A—C1A—C6A—C5A	−0.6 (2)
C2—C3—C4—C5	1.7 (2)	C2A—C3A—C4A—C5A	−1.8 (2)
C3—N1—C7—C8	−92.09 (19)	C3A—N1A—C7A—C8A	−83.25 (19)
C3—N1—C9—C10	−81.0 (2)	C3A—N1A—C9A—C10A	−98.73 (19)
C3—C4—C5—C6	−1.0 (2)	C3A—C4A—C5A—C6A	0.9 (2)
C4—C5—C6—C1	−0.6 (2)	C4A—C5A—C6A—C1A	0.3 (2)
C6—C1—C2—C3	−0.6 (2)	C6A—C1A—C2A—C3A	−0.4 (2)
C7—N1—C3—C2	−173.91 (15)	C7A—N1A—C3A—C2A	−176.56 (15)
C7—N1—C3—C4	6.4 (2)	C7A—N1A—C3A—C4A	3.0 (2)
C7—N1—C9—C10	92.77 (18)	C7A—N1A—C9A—C10A	87.86 (18)
C9—N1—C3—C2	−0.5 (2)	C9A—N1A—C3A—C2A	10.4 (2)
C9—N1—C3—C4	179.73 (15)	C9A—N1A—C3A—C4A	−169.98 (15)
C9—N1—C7—C8	94.20 (18)	C9A—N1A—C7A—C8A	90.25 (18)

### Hydrogen-bond geometry (Å, º)

**Table:**

*D*—H···*A*	*D*—H	H···*A*	*D*···*A*	*D*—H···*A*
O1—H1···O1*A*	0.86 (1)	1.92 (1)	2.7445 (16)	160 (2)
O1*A*—H1*A*···O1^i^	0.86 (1)	1.91 (1)	2.7599 (16)	170 (2)

Symmetry code: (i) −*x*+1, −*y*+1, −*z*+1.
