# Supplementary figures and images for: Crystal structure of 3-(di­ethyl­amino)­phenol
Source: Acta Crystallogr E Crystallogr Commun. 2015 Dec 24;71(Pt 12):o1075. doi: 10.1107/S2056989015024226 (PMC4719986; doi:10.1107/S2056989015024226)

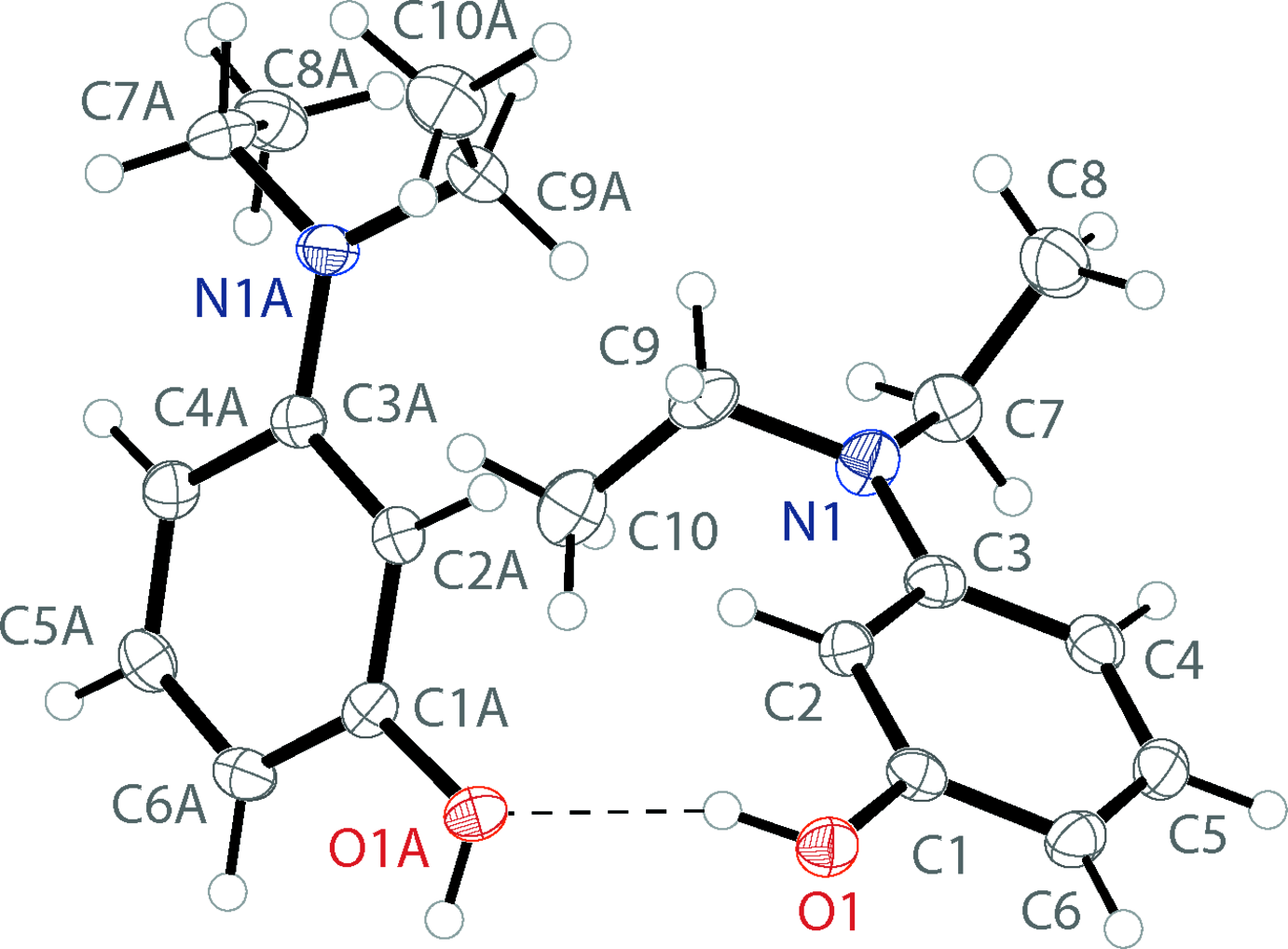

Supplement: Supplementary file 4 [file e-71-o1075-fig1.tif]

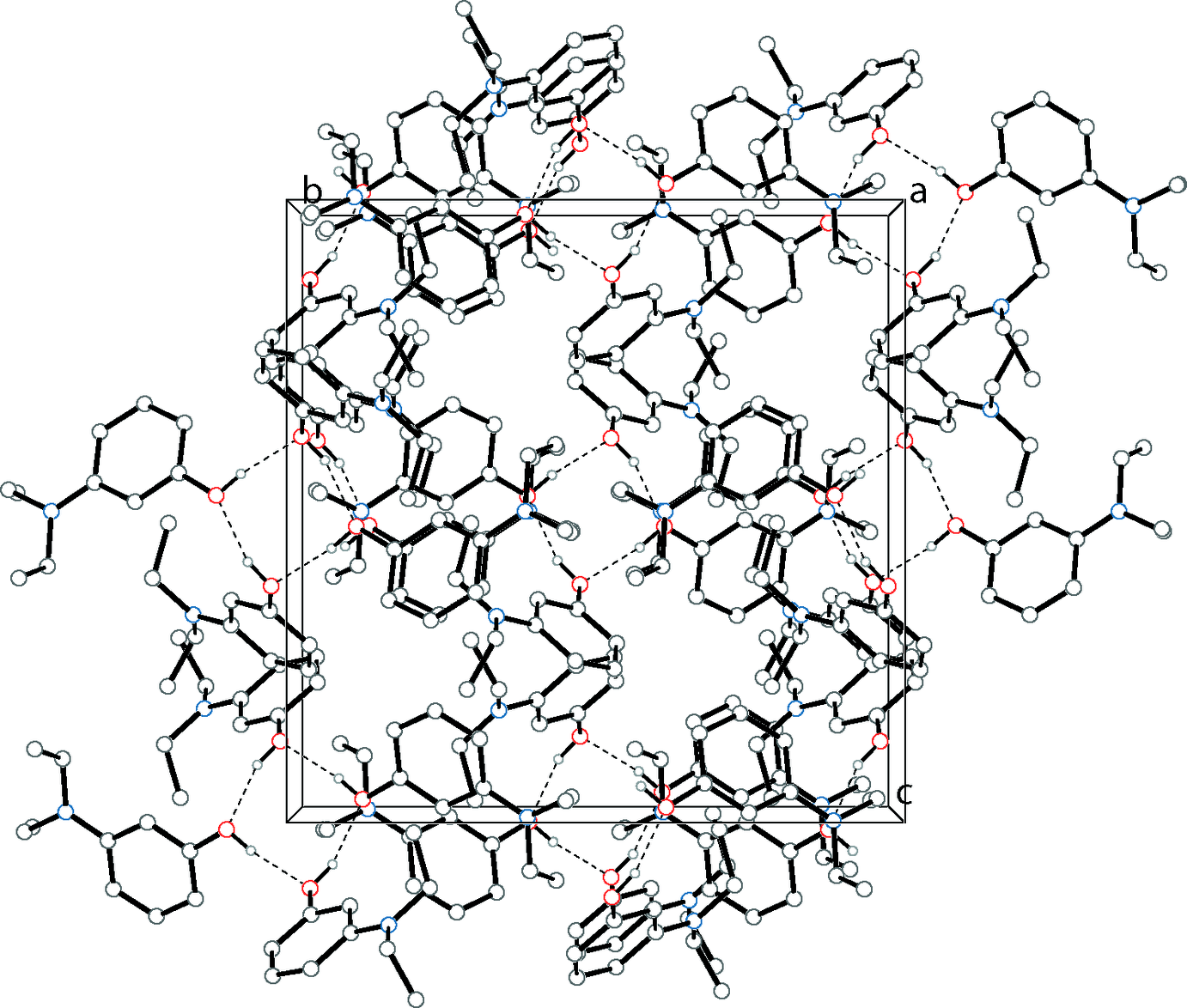

Supplement: Supplementary file 5 [file e-71-o1075-fig2.tif]
